# Methotrexate conjugated gold nanoparticles improve rheumatoid vascular dysfunction in rat adjuvant-induced arthritis: gold revival

**DOI:** 10.1007/s10787-022-01104-w

**Published:** 2022-12-08

**Authors:** Salma T. Rafik, Teshreen M. Zeitoun, Thanaa I. Shalaby, Mervat K. Barakat, Cherine A. Ismail

**Affiliations:** 1 Department of Clinical Pharmacology, Faculty of Medicine, Alexandria University, Alexandria, Egypt; 2 Department of Histology and Cell Biology, Faculty of Medicine, El-Moassat Medical Hospital, Alexandria University, Alexandria, Egypt; 3 Department of Medical Biophysics, Medical Research Institute, Alexandria University, Alexandria, Egypt

**Keywords:** Rheumatoid arthritis, Endothelial dysfunction, Atherosclerosis, VCAM-1, α-SMA

## Abstract

Endothelial vasomotor dysfunction and accelerated atherosclerosis encompass the features of rheumatoid vascular dysfunction (RVD), increasing cardiovascular morbidity and mortality among rheumatoid arthritis (RA) patients. Methotrexate, among DMARDs, effectively reduces cardiovascular events, but its non-selectivity together with its pharmacokinetic variability often limit drug adherence and contribute to its potential toxicity. Thus, methotrexate was conjugated to gold nanoparticles (MTX/AuNPs) and its effect on RVD in rats’ adjuvant-induced arthritis was evaluated. A comparative study between MTX/AuNPs, free MTX, and AuNPs treatments on joint inflammation, vascular reactivity and architecture, smooth muscle phenotype, systemic inflammation, and atherogenic profile was done. Since MTX/AuNPs effect was superior, it appears that conjugation of MTX to AuNPs demonstrated a synergistic action. MTX immunomodulatory action combined with AuNPs anti-atherogenic potential yielded prompt control of whole features of RVD. These findings highlight the usefulness of nanoparticles-targeted drug-delivery system in refining rheumatoid-induced vascular dysfunction treatment and reviving gold use in RA.

**Supplementary Information:**

The online version contains supplementary material available at 10.1007/s10787-022-01104-w.

## Introduction

An overwhelming link between rheumatoid arthritis (RA) and cardiovascular disease (CVD) is evidenced by excess CV morbidity and mortality among RA patients. Several mechanisms orchestrate this link, including shared inflammatory mediators, altered composition and function of lipoproteins, oxidative stress, and a subsequent targeted immune response activation. These mechanisms interplay to evoke a rheumatoid-induced vascular dysfunction increasing CV risk (Argnani et al. 2021). In RA, a chronic inflammatory process is established in the vascular wall, which procures to a self-perpetuating cycle promoting chronic endothelial vasomotor dysfunction and accelerated atherosclerosis that are considered independent determinants of CVD (Castañeda et al. 2016). These two major features encompass the main picture of rheumatoid vasculitis, which is arguably the most serious extra-articular systemic manifestation of RA with substantial morbidity and mortality despite the aggressive use of disease-modifying therapies. The onset of overt vasculitis symptoms is substantially late during the course of RA despite the premature onset of the underlying pathology that can affect many organs (Tanasescu et al. 2009).

In the last updated edition, the European league against rheumatism (EULAR) recommendations emphasized the importance of optimum control of disease activity to lower CVD risk in rheumatoid patients (Agca et al. 2017). Based on the current understanding of the major role of inflammation on CV risk in RA (Castañeda et al. 2016; Radic et al. 2013), Yu et al. (2018) claimed that CV risk may be modulated by controlling inflammation in RA.

Unfortunately, the disease-modifying anti-rheumatic drugs (DMARDs) exert differential effect on CV risk besides a critical focus on their toxicity-related burden on patients’ health (Lopez-Olivo et al. 2014). Indeed, England et al. (2018) concluded that the aggressive use of DMARDs together with a prompt management of CV risk factors are essential to reduce the substantial burden posed by this common comorbidity. They noticed that among all drugs treating RA, only methotrexate (MTX) and TNF inhibitors reduce all CV events, including myocardial infarction and cerebrovascular accidents. MTX’s relative risk reduction was even higher than that of TNF inhibitors.

Nevertheless, the non-selective activity of MTX together with the variability in its pharmacokinetics often limit dose escalation, drug adherence, and likely contribute to its potential toxicity. Over the past decades, efforts have been made to modify the pharmacokinetic behavior of MTX to enhance its organ targeting ability and tolerability and overcome its cellular drug resistance. In this regard, the most successful approach, thus far, was to design and constitute the drug-delivery system for MTX. Thus, a targeted therapy approach, for example nanocarriers, that allows a selective drug delivery to the target sites with sufficient effective concentrations avoiding high dosing becomes highly desirable in RA therapy (Lopez-Olivo et al. 2014; Pham 2011).

Indeed, Gold nanoparticles (AuNPs), as an effective example of nanocarriers, have been extensively used in medicine and recently have been involved in controlled drug-delivery systems (DDS), particularly in cancer (Kong et al. 2017). The ease of synthesis and the unique optical, electronic, and biochemical properties of AuNPs make them ideal candidates for translation into clinical therapeutics (Sibuyi et al. 2021).

Of interest, spherical AuNPs (AuNSs) are one of the most widely used gold nanostructures in drug-delivery applications. The AuNSs possess useful features, such as size- and shape-related optoelectronic properties, large surface to volume ratio increasing drug payload, excellent biocompatibility, and low toxicity (Kong et al. 2017). Although gold may have fallen out of favor as a mainstream therapeutic agent in a wide variety of rheumatic diseases, its use in NPs is set to revive its application in medical care (Bansal et al. 2020). The coupling of MTX to AuNPs has already shown efficacy in an adjuvant model of RA as a joint disease, but it is not yet studied in rheumatoid-induced vascular dysfunction and CV risk (Chen et al. 2013).

Therefore, the present study aimed to determine the modulatory effect of methotrexate-conjugated to gold nanoparticles (MTX/AuNPs) on RA-associated vascular dysfunction in complete Freund's adjuvant (CFA)-induced arthritis model in Wistar rats.

## Methods

### Animals

The present study was conducted on 65 female Wistar albino rats (including 12 rats used in toxicity study) weighing 150–200 g. Animals were purchased from the animal house of the Faculty of Medicine, Alexandria University. The rats were housed under standard conditions of light and temperature with free access to food and water in accordance with the ARRIVE guidelines for animal care. All experimental procedures were approved and performed in compliance with the guidelines of the Local Ethics Committee of Alexandria, Faculty of Medicine, University of Alexandria. Protocol approval number: 0104517.

### Chemicals

Methotrexate was supplied by Techno Pharma–Egypt. Gold salt: chloroauric acid.

(HAuCl_4_.3H_2_O, 99.9%, Trisodium citrate dihydrate (Na_3_C_6_H_5_O_7**.**_2H_2_O, 1%), Complete Freund’s Adjuvant (CFA), Phosphate Buffered Saline (PBS) PH = 7.4 and Acetylcholine (ACh) (Sigma-Aldrich—Egypt). Anti-Alpha-smooth muscle actin antibody: (Affymetrix ebioscience Inc—USA), Norepinephrine (NE): (MYLAN—USA) and Krebs solution: (NaCl; 118.3, KCl; 4.69, CaCl_2_; 1.87, MgSO_4_; 1.20, K_2_HPO_4_; 1.03, NaHCO_3_; 25.0, and glucose 11.1) (Elnasr company—Egypt).

### Synthesis, characterization, and quantification of gold and MTX loaded gold nanoparticles

#### Synthesis of gold and MTX loaded gold nanoparticles

Gold nanoparticles (AuNPs) of about 11–20 nm in size were prepared by the citrate reduction of chloroauric acid (HAuCl_4_), as described by Turkevich and Frens with modification (Kimling et al 2006). After boiling of an aqueous solution of HAuCl_4_.3H_2_O (1 mM, 5 ml), it was mixed with trisodium citrate Na_3_C_6_H_5_O_7**.**_2H_2_O (10 ml, 1 wt%) and left to boil. When the solution had turned deep red, it was allowed to cool at room temperature with continuous stirring. To eliminate the free citrate, the solution was subjected to high-speed centrifugation and then the pellet was suspended three times in PBS at pH 7.4. For drug loading, MTX solution (1.5 mg/ml) was incubated with AuNPs in the ratio of 3:4, respectively at 37 °C for 48 h, then the mixture was subjected to centrifugation at 15,000 rpm for 20 min and the pellet was washed and dispersed in PBS. Further determination of the loading efficiency of MTX on AuNPs was done by HPLC (Waters UV/Visible detector 2489—Alexandria University). The MTX uptake by AuNPs was determined by calculating the difference between the initial and residual amounts of MTX in the preparing solution known as loading efficiency, as follows (Chen et al. 2013):

$${\text{LE}}\% = \frac{{\left( {{\text{CT}} - {\text{CF}}} \right)}}{{{\text{CT}}}} \times 100$$

where, LE is the loading efficiency, CT is the total MTX concentration, CF is the free MTX concentration

#### Physiochemical characterization of the prepared nanoparticles

##### Zeta potential measurements

The hydrodynamic size, polydispersity index (PDI) and zeta potential of gold nanoparticles before and after loading with MTX were measured using a Nano Zetasizer particle analyser (Malvern, UK). The nano zeta sizer measures the diameter of the particles using the dynamic light scattering (DLS) technique. This involves detection of the scattered light from particles suspended in an aqueous solution at 25 °C (Eidi et al. 2012).

##### Ultraviolet–visible absorption (UV/VIS) spectroscopic measurements.^(16)^

The surface Plasmon resonance (SPR) of AuNPs and MTX/AuNPs was monitored by UV/Vis absorption spectroscopy (UV5-220—ThermoFisher Scientific spectrophotometer – Germany), at a resolution of 1–2 nm. The SPR depicts the size and distribution of nanoparticles helping to monitor the MTX loading on AuNPs (Oza et al. 2012).

##### Fourier Transform Infrared Spectroscopy (FTIR) measurements

FTIR spectra of AuNPs and MTX/AuNPs were recorded on a Shimadzu FTIR-8400S (Tokyo – Japan) at a resolution of 4 cm^−1^ over the range of 400–4000 cm^−1^ on potassium bromide (KBr) pellets. The produced absorbance spectrum shows the wavelengths that the sample absorbs revealing details about the sample molecular structure and chemical bond functional groups (Smith 2011).

##### Transmission electron microscopy (TEM)

The size and shape of the prepared NPs were detected using TEM (Jeol 100 CX, Tokyo—Japan). A small drop of the AuNPs solution was placed onto TEM grids coated with a thin carbon film and allowed to evaporate. Then, digital pictures of several locations on the grid were taken (Waseda and Muramatsu 2004).

### Subacute in vivo toxicity study

Since AuNPs can accumulate in secondary organs, the aim of this toxicity study was to detect their biocompatibility and potential organ toxicity and to determine the safety of the chosen AuNPs dose. This study was done on 12 female Wistar albino rats randomly divided into 2 equal groups. First group administered daily 500 µg/Kg of the synthesized citrated AuNPs subcutaneously (*sc*) for 4 weeks versus a normal control group that received daily *sc* PBS. Rats were closely observed daily for some pre-set human endpoints, including any signs of morbidity, weight reduction, abnormal behaviors, disturbed bowel habit, or impaired ambulation. Survival rate and coagulation time were followed throughout the 2 weeks. Afterward, rats were sacrificed, and a gross necropsy was blindly performed for kidney, heart, liver and aorta, together with histological and hematological studies to identify possible target organ(s) toxicity (Akhila et al. 2007).

### Experimental design and induction of arthritis

After acclimatization, animals were initially divided into 2 groups: Normal control (10 rats) and adjuvant-induced arthritis (AIA) group (40 rats). For induction of arthritis on day zero, CFA was mixed with PBS (1:1 v/v). Immediately before injection, the mixture was shaken carefully, and 0.3 ml was injected intradermally at the base of the tail, while normal control rats were injected with PBS (Gutierrez-Rebolledo 2015). Ankle joint diameters (anteroposterior and mediolateral diameters) were measured every 2 days using digital caliper to check for the onset of arthritis. Upon development of arthritis, adjuvant-induced arthritis (AIA) group was randomly divided into 4 subgroups according to the treating agent: AuNPs (500 µg/kg *sc* in the dorsal flank); MTX (40 µg/kg intraperitoneally, *ip*); MTX-AuNPs (20 µg/kg *sc*); vehicles (PBS 1 ml/100 g *bw sc* and 1 ml Normal saline *ip)* for both diseased, as well as for normal control (Chen et al. 2013).

### Sampling and biochemical and histological assessments

On the 29^th^ day of the experiment, the animals were anesthetized by inhalation of isoflurane and blood samples were collected by heart puncture, centrifuged at 3000 rpm for 10 min and stored at -20˚C for assessment of serum VCAM-1 (ELIZA kit, eBioscience Inc—USA) (dos Santos et al. 2018), CRP (hsELISA kit, Chemux bioscience, Inc—USA) (McGill and Gronowski 2018) and lipid profiles using colorimetric kit, Linear chemicals—Spain) (Allain et al. 1974; Grove 1979; Bucolo and David 1973; Knopfholz et al. 2014). After sacrificing animals by profound anesthesia, both thoracic aortae, femoral arteries, ankle joints were dissected, fixed in formol saline 10%, processed, sectioned, and stained with haematoxylin and eosin (H&E). The extent of ankle joint inflammation was determined using a semi-quantitative modified composite graded scale (Eissa et al. 2016). Furthermore, the dissected vessels were stained with orcein (Drury and Wallington 1980) and immunohistochemically with alpha-smooth muscle actin antibody (Immunodetector HRP/DAB detection system, Bio SB, Inc—USA) that was subjected to morphometric study (Chen et al 2016). All sections were assessed blindly.

### Biological isometric tension study of the aorta

Contraction-relaxation response curves of the norepinephrine (NE) pre-contracted isolated aortic rings (Number = 6/ group) to ACh were plotted using ADInstruments PowerLab 8/35 data acquisition system (Model No PL3508/P, ADInstruments Pty Ltd, Castle Hill-Australia); as a tool for assessment of endothelial dysfunction. A section of the thoracic aorta was carefully dissected, isolated, and immediately put in a freshly prepared Krebs solution. The clean aortae were then cut into rings of approximately 3–5 mm width and each ring was gently attached to a force sensitive isometric transducer (Model MLT0202, AD Instruments) and immersed in a 20 ml organ bath chamber filled with Kreb’s solution. The solution was continuously aerated with carbogen (95% O_2_, 5% CO_2_ mixture) and maintained at 37 °C. Tissues were allowed to equilibrate for 1 h after applying a passive tension of 2 g with a frequent wash every 15 min. Following equilibration, cumulative concentrations (10^–7^–10^–4^ mol/l) of NE were added to obtain a contraction dose–response curve (constrictor response). Then, cumulative concentration response curves for the relaxant effect of ACh (10^–7^–10^–3^ mol/l) on the NE pre-contracted rings were recorded. Responses were expressed as a percentage of relaxation through reduction of NE-peak response (Jespersen et al. 2015).

### Statistical analysis

Data were subjected to analysis using Graph Pad Prism v.7.0 software package. One-way Analysis of Variance (ANOVA) test was utilized followed by Tukey’s or Dunn’s multiple comparison tests for parametric and non-parametric data, respectively. Concentration–response curves to NE and ACh were compared by two-way ANOVA for repeated measures followed by Tukey’s multiple comparison test. Unpaired t-test was utilized for comparison between two groups. A value of *P* < 0.05 was considered statistically significant. Results were expressed as Mean ± SEM.

## Results

### Characterization of AuNPS and MTX-AuNPs

**Fig. 1:**
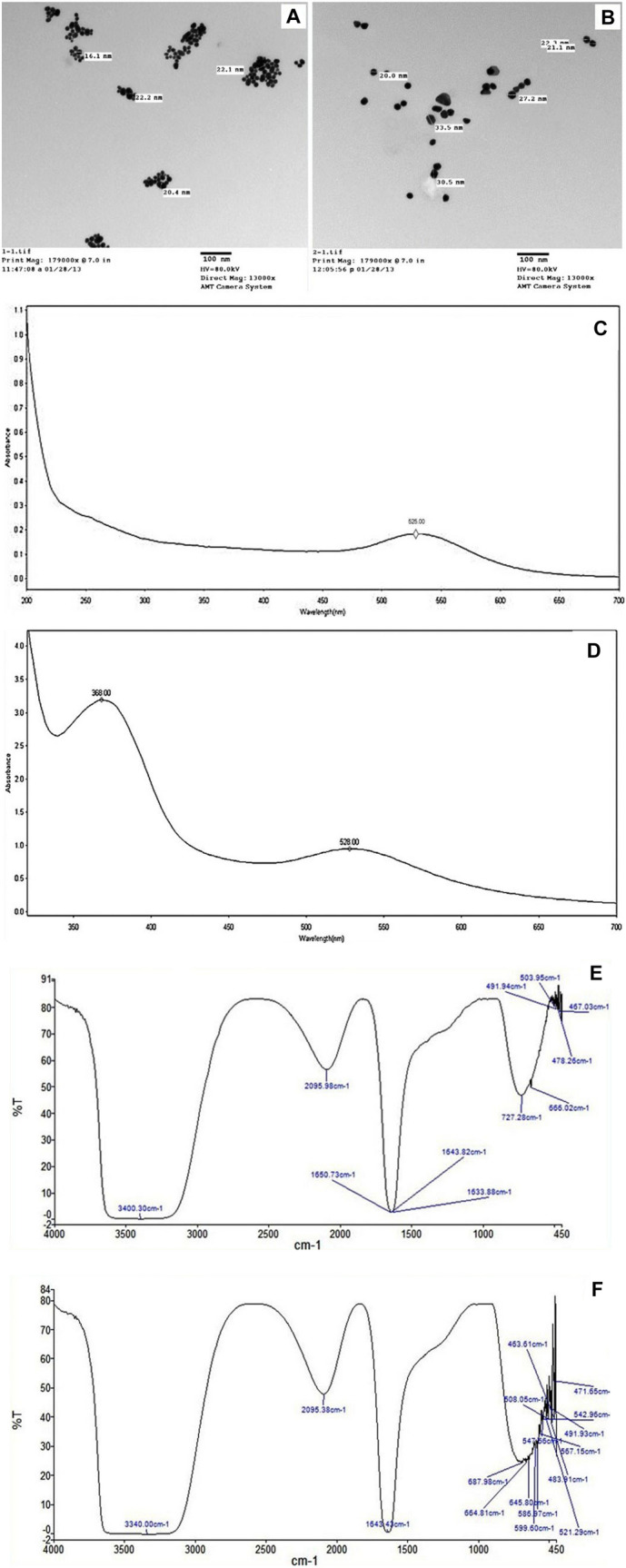
Characterization of AuNPs & MTX/AuNPs. **A** & **B** TEM micrographs of AuNPs and MTX/AuNPs, respectively. **C** & **D** Absorption spectra of prepared AuNPs before and after conjugation with MTX. (**E** & **F**) FTIR analysis of AuNPs before and after conjugation with MTX

The morphology of the prepared nanoparticles was revealed using Transmission Electron Microscopy (TEM). TEM examination revealed that the particles were spherical in shape and the size range of AuNPs and MTX/AuNPs was 16–22 and 20–33 nm, respectively. No particle aggregation was noted (Fig. 1 A, B). The hydrodynamic particle size, polydispersity index (PDI) and zeta potential *(ζ)* of both AuNPs and MTX/AuNPs were determined using Zetasizer (Table 1, Fig. S1 A, B). The UV–VIS spectroscopy revealed that the surface Plasmon band of prepared AuNPs has a peak absorbance at 525 nm. Whereas, after MTX conjugation, another peak appeared at 368 nm, which reflects the free MTX and the AuNPs peak Plasmon absorption was shifted from 525 to 528 nm (Fig. 1 C, D). The 3 nm shift in the AuNPs peak Plasmon absorption is due to a change in local dielectric constant around the AuNPs, as a result of MTX adsorption. To further validate the loading of MTX on AuNPs, the FTIR spectrum of AuNPs was compared to AuNPs conjugated to MTX (Fig. 1 E, F). After MTX binding to the AuNP, the broad band in the range of 3200–3600 cm^−1^ is due to the presence of surface bound –H3-N, –OH functionalities on the nanoparticle surface. This band overlapped with the – OH stretching vibration of MTX. The appearance of hydrogen bonds at 1643 cm-^1^ between the cytostatic and the carrier can be attributed to the bending vibration of N–H and stretching vibration of C = O, C-H, O–H. This energy shift was in line with the shift in the absorption bands of UV–VIS spectrum and both denote the interaction of MTX with the nanoparticle molecules.

**Table 1:** Characterization of AuNPs & MTX/AuNPs by zeta sizer

Nanoparticles parameters	Particle Size (nm)	Zeta potential (mV)	Polydispersity index (PDI)
AuNPs	25.4	– 34.0	0.42
MTX/AuNPs	35.4	– 25.0	0.40

The average hydrodynamic diameter of the prepared AuNPs was 25.4 nm and the PDI was 0.42. After conjugation with MTX, the mean diameter was increased to be 35.4 nm and the PDI became 0.40. Zeta potential measurements showed a negatively charged surface potential of the non-conjugated AuNPs with approximately – 34.0 mV (which is attributed to the presence of citrate) that decreased to – 25.0 mV after conjugation with MTX, confirming its conjugation onto the surface of AuNPs

### Toxicity study

Survival rate, hepatic and renal functions tests, and bleeding, coagulation, and prothrombin time showed non-significant difference between rats injected with 500 µg/Kg of citrated AuNPs and normal control throughout the 4 weeks (data not shown). Blindly performed gross necropsy and histological studies of dissected organs from rats injected with citrated AuNPs were nearly comparable to that of normal control.

### General condition & signs of arthritis

The general condition of all treated and non-treated-CFA injected rats was satisfactory throughout the study, and they gained weight comparable to normal control. After about 14 days from the beginning of the study, rats injected with CFA started to show signs of inflammation in diverse joints that were manifested by the significant increase in the combined ankle joint diameter by 6.4 + 0.5 mm^2^ (*P* < 0.0001, Unpaired *t*-test) (Fig. S2). Some rats, especially in the non-treated diseased rats, developed tail inflammation and difficulty in locomotion.

### Biochemical analysis

#### Serum VCAM-1 and CRP (ng/ml)

**Fig. 2:**
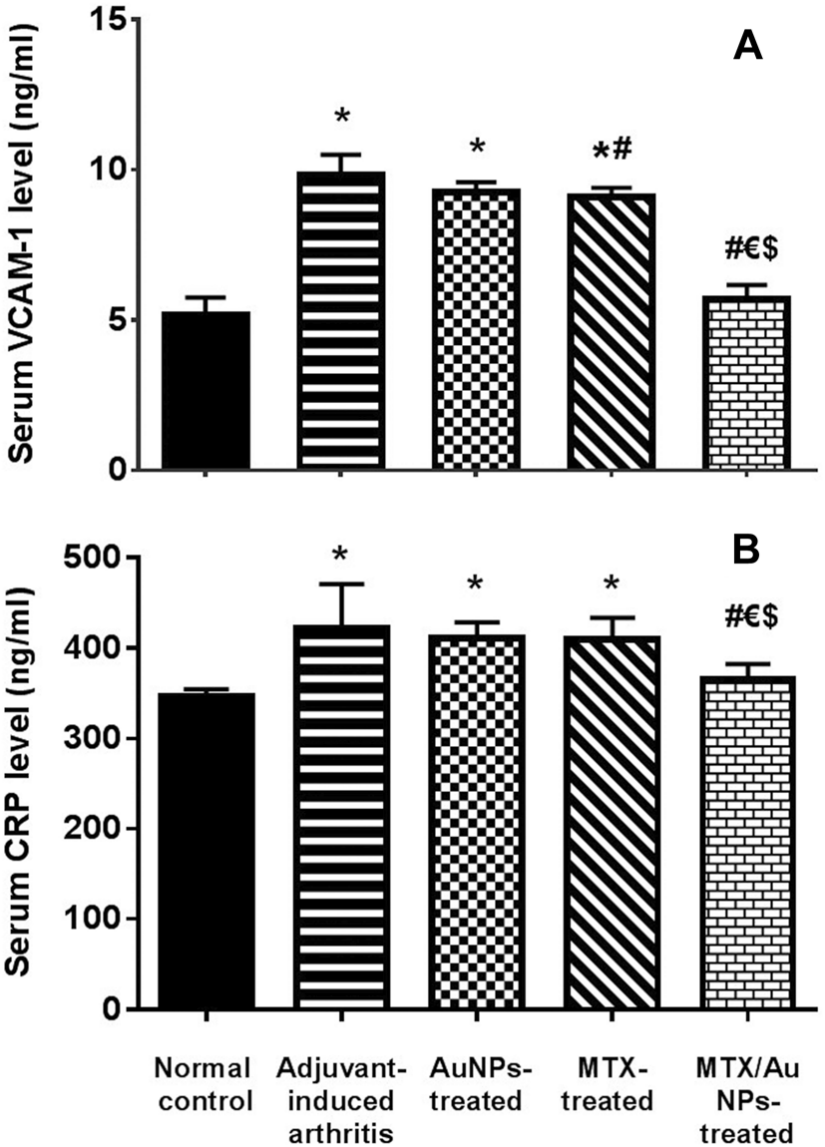
Effect of MTX/AuNPs treatment in adjuvant-induced arthritis on serum VCAM-1 **A** and CRP levels **B**. MTX: methotrexate, MTX/AuNPs: methotrexate conjugated to AuNPs. Number of rats/group = 10. Data are expressed as means ± SEM. *p* < 0.05 *: Significant difference versus normal control, # Significant difference versus adjuvant-induced arthritis group, € Significant difference versus AuNPs-treated rats, $ Significant difference versus methotrexate-treated rats

Injection of CFA in the AIA rats induced a significant increase in serum VCAM-1 and CRP levels compared to normal control indicating a chronic inflammatory state (Fig. 2 A, B, respectively). Daily administration of MTX/AuNPs in AIA rats for 14 days showed a significant decrease in both serum VCAM-1 and CRP levels versus AIA rats, and both free MTX- and non-conjugated AuNPs-treated rats. The administered dose of free MTX succeeded in significantly decrease only serum VCAM-1 versus non-treated AIA rats.

#### Lipid profiles

##### Serum total cholesterol, HDL-C, LDL-C & triglycerides (ng/ml)

**Fig. 3:**
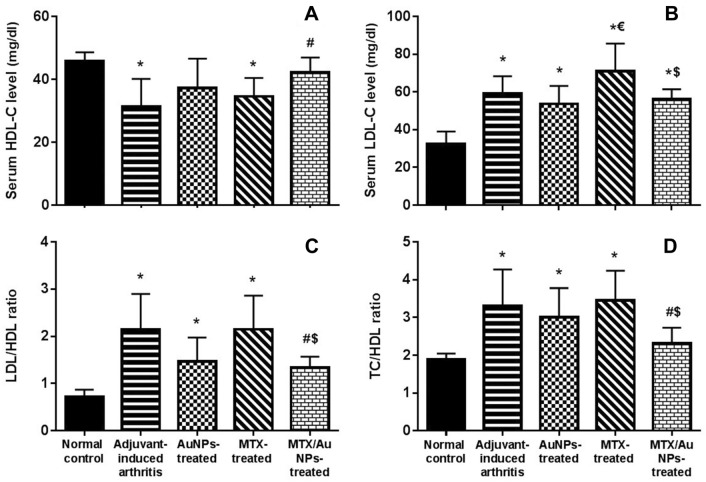
Effect of MTX/gold nanoparticles treatment in adjuvant-induced arthritis on serum lipid profiles: **A** HDL-C: high density lipoprotein-cholesterol, **B** LDL-C: low density lipoprotein-cholesterol, **C** LDL/HDL ratio, **D** TC/HDL ratio: total cholesterol/HDL ratio. MTX: methotrexate, MTX/AuNPs: methotrexate conjugated to AuNPs. Number of rats/group = 10. Data are expressed as means ± SEM. *p* < 0.05 * Significant difference versus normal control, # Significant difference versus adjuvant-induced arthritis group, $ Significant difference versus methotrexate-treated rats

While injection of CFA in the AIA rats did not induce any significant changes in serum total cholesterol (TC) and triglycerides, it induced a significant decrease in HDL-C and a significant increase in LDL-C levels versus normal control. Daily administration of MTX/AuNPs showed a significant increase in HDL-C level with a non-significant decrease in LDL-C level versus non-treated AIA group, yet LDL-C level was significantly lower than free MTX treatment. Strange enough, free MTX non-significantly increased HDL-C and significantly also raised LDL-C level versus non-treated AIA rats. Interestingly, non-conjugated AuNPs treatment non-significantly increased HDL-C and decreased LDL-C levels versus non-treated AIA group denoting a potential anti-atherogenic action (Fig. 3 A, B and Fig. S3).

##### Atherogenic ratios (Total cholesterol/HDL ratio & LDL/HDL ratio)

These ratios TC/HDL and LDL/HDL represent an atherogenic index, which is an important prognostic marker for CVD that correlates with disease activity. Both ratios were significantly increased by CFA injection in non-treated AIA rats versus normal control suggesting an observed high atherosclerosis risk. They were significantly reduced by MTX/AuNPs treatment versus both non-treated AIA and free MTX treated rats. Free MTX treatment non-significantly increased both ratios, while again they were non-significantly reduced by non-conjugated AuNPs treatment (Fig. 3 C, D).

### In-vitro vascular reactivity study

**Fig. 4:**
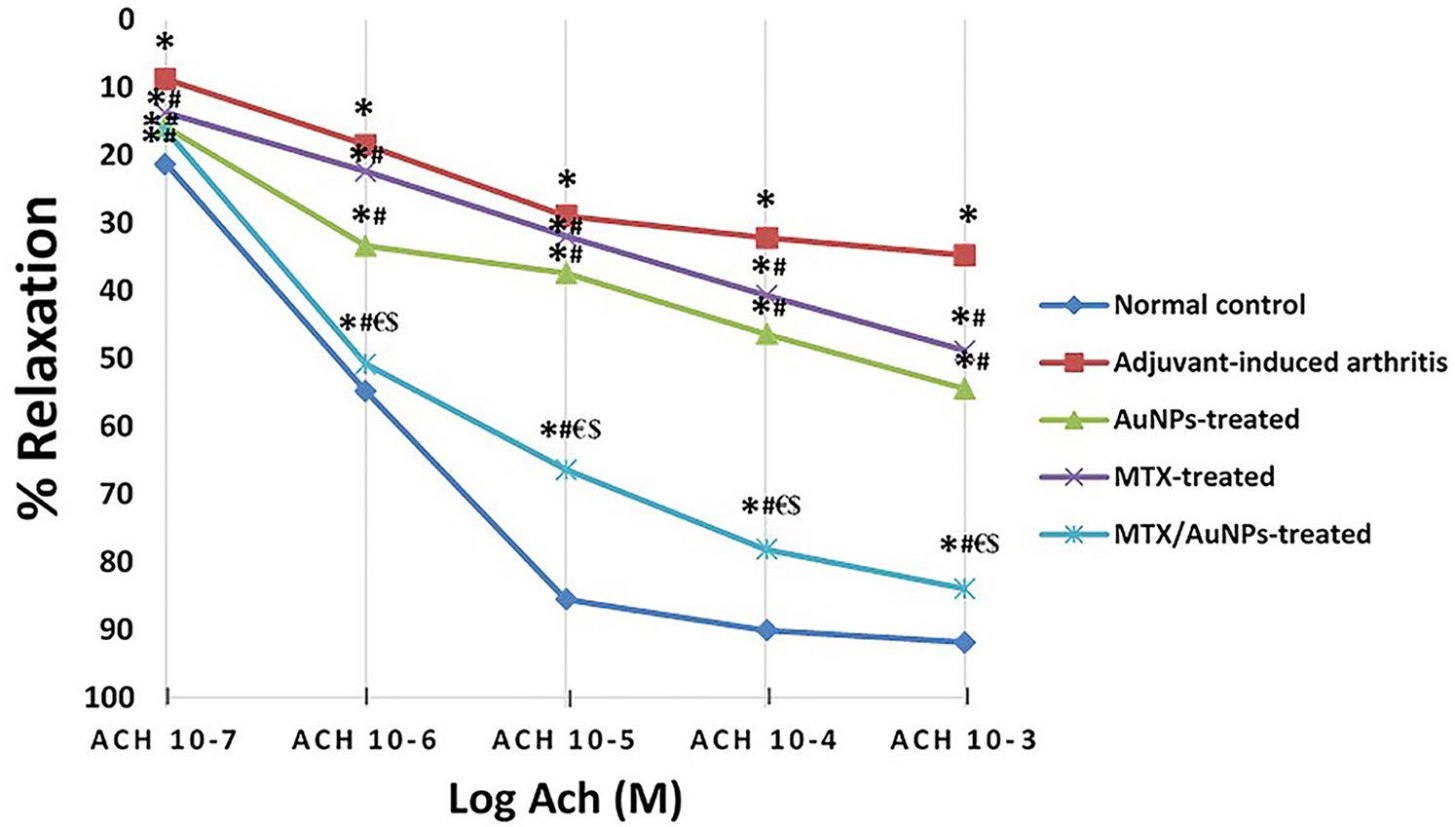
Percent relaxation of NE pre-contracted aortic rings in response to cumulative doses of ACh (10^–7^–10^–3^ mol/l) revealed a significantly enhanced ACh-induced relaxant response by MTX/AuNPs. MTX: methotrexate, MTX/AuNPs: methotrexate conjugated to AuNPs, ACh: acetylcholine. Data are expressed as means ± SEM for *n* = 6. *p* < 0.05, by two-way ANOVA for repeated measures. * Significant difference versus normal control, # Significant difference versus adjuvant-induced arthritis group, € Significant difference versus AuNPs-treated rats, $ Significant difference versus methotrexate-treated rats

Cumulative concentrations of NE (10^–6^ to 10^–4^ mol/L) induced vasoconstriction in a dose-dependent manner in aortae isolated from the normal control, with maximum contraction achieved at 10^–4^ mol/L. The contraction response of the non-treated AIA aortae was significantly higher versus normal control and all other treated groups (Fig. S5). The maximally contracted aortic rings of normal control rats showed a dose-dependent relaxant response to cumulative doses of ACh (10^–7^ to 10^–3^ mol/L). A marked significant reduction in ACh-induced relaxation in maximally contracted rings of the non-treated AIA rats versus those of normal control rats. Administration of MTX/AuNPs attained a significant improvement of vascular reactivity demonstrated by a significantly enhanced ACh-induced relaxant response versus those of the non-treated AIA rats and both free MTX- and AuNPs-treated rats (Fig. 4).

### Histological assessments

#### Haematoxylin and eosin stain

**Fig. 5:**
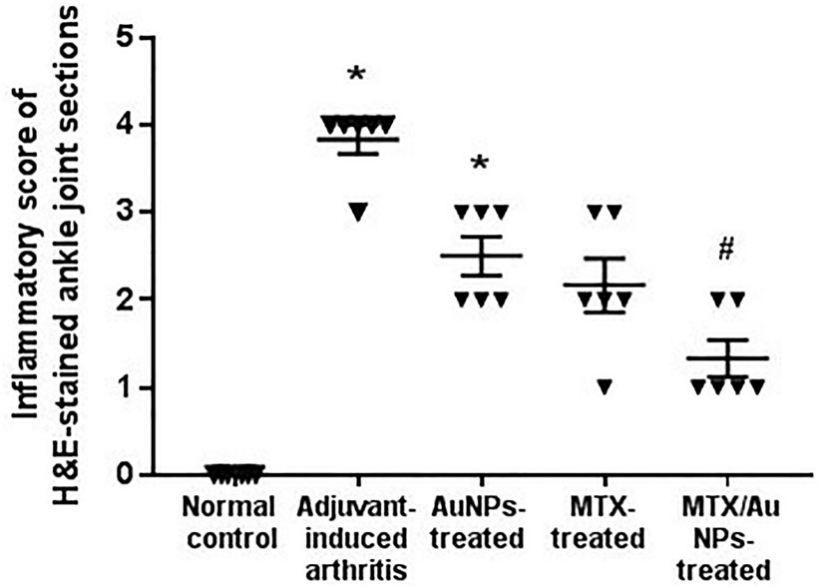
Semi-quantitative inflammatory score of H&E-stained tissue sections of rats' ankle joints confirmed the significant attenuation of joint inflammation by MTX/AuNPs treatment versus non-treated AIA rats. *MTX* methotrexate, MTX/AuNPs: methotrexate conjugated to AuNPs, ACh: acetylcholine. Data are expressed as medians. *p* < 0.05 *: Significant difference versus normal control, # Significant difference versus adjuvant-induced arthritis group

**Fig. 6:**
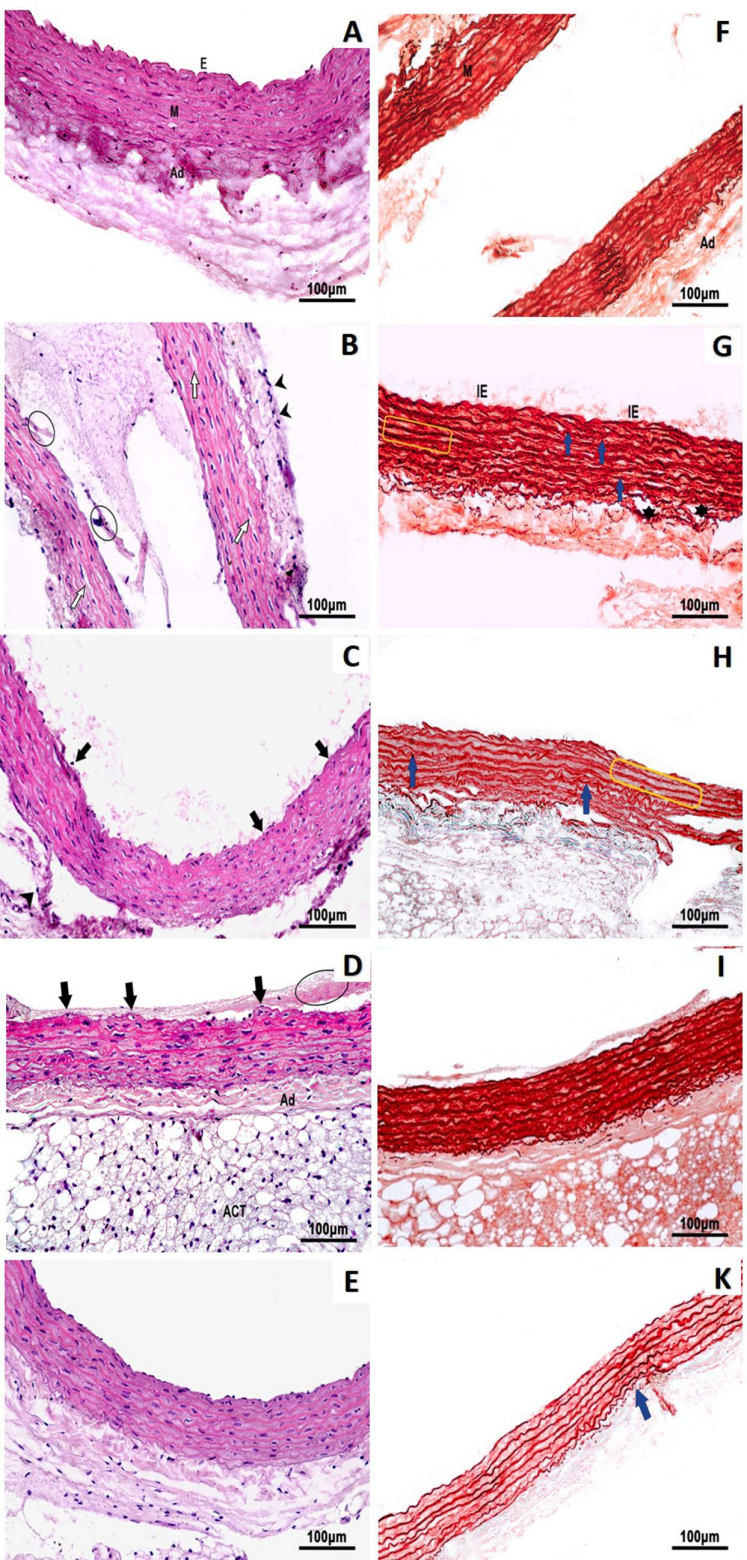
Representative photomicrographs of rats' aortae tissue sections showing in (**A**–**E)**, **H**&**E** stain and in (**F**–**K**) Orcein stain of normal control, non-treated AIA, AuNPs-, MTX-, and MTX/AuNPs-treated rats, respectively. *E* endothelial cells, *M* tunica media, *Ad* adventitia, *IE* internal elastic lamella, *ACT* adipose connective tissue. Note, cellular debris in lumen (circle), focal areas of endothelial loss (black arrow), multiple edematous areas with loss of staining in M (white arrow), inflammatory cellular infiltration of adventitia (arrowhead), disorganization of elastic lamellae (blue arrow), regional flattening of elastic lamellae (yellow rectangle), frequent areas of wide separation of elastic sheets (⋆)

Ankle joint examination of the non-treated AIA rats revealed features of arthritis in the form of thickened synovial membrane with congested blood vessels and edema, associated with narrow joint space and irregular surfaces of the articular cartilage. Whereas those of the MTX/AuNPs and free MTX showed a relative regression of inflammatory features with wider joint space and smoother cartilaginous surface (Fig. S6 A-F). Statistical analysis of histological semi-quantitative inflammatory score confirmed the improvement of arthritis features by all treatments versus non-treated AIA rats, being only significant with MTX/AuNPs treatment (Fig. 5). Both aorta and femoral arteries of the non-treated AIA rats revealed features of vascular dysfunction in the form of intimal irregularity with focal loss and the lumen was filled with inflammatory cells and sloughed endothelial tissue. The tunica media of the aorta showed multiple oedematous areas, while that of the femoral artery was thinned out and both revealed a thickened adventitia infiltrated by inflammatory mononuclear cells. The femoral artery was surrounded with several smaller vessels with disorganized irregular lumens. Treatment with MTX/AuNPs restored vascular architecture expressing almost intact endothelium with normal concentric elastic membranes alternating with smooth muscles fibers in tunica media and lack of inflammatory cells infiltration. The improvement observed in free MTX vascular structure was less evident than in MTX/AuNPs-treated rats, yet much improved than with non-conjugated AuNPs treatment. Both MTX/AuNPs and free MTX treatment abolished the neovascularization around the femoral artery observed in the non-treated AIA rats (Fig. 6 A–E for aorta and Fig. S7 A–E for femoral).

#### Orcein stain

Orcein stain selectively demonstrates elastic fibers in vascular tissue sections. The elastic lamellae in non-treated AIA aortae were disorganized and destructed with areas of wide separation, whereas almost intact elastic lamellae were seen in both MTX/AuNPs and free MTX aortae (Fig. 6*F*-*K*). Femoral sections of the MTX-AuNPs-treated rats revealed more prominent internal and external elastic laminae with normal thickness than rats treated with free MTX and non-conjugated AuNPs (Fig. S7 F-K).

#### Immunohistochemical staining of α-SMA

**Fig. 7:**
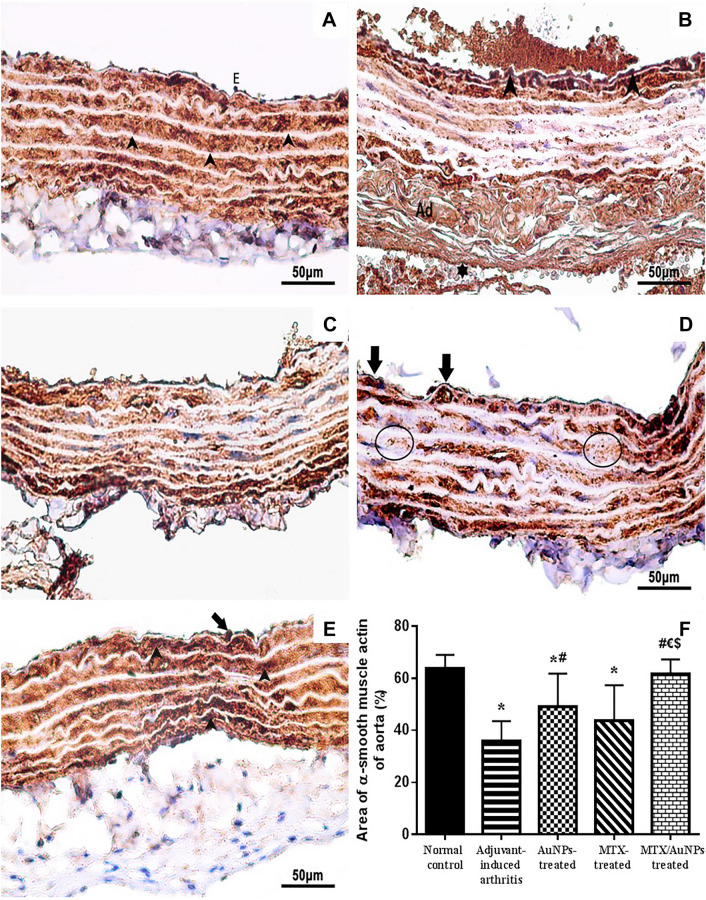
Representative photomicrographs of immunohistochemically stained rats' aortae tissue sections with alpha-smooth muscle actin antibody (HRP/DAB) of normal control, non-treated AIA, AuNPs-, MTX-, and MTX/AuNPs-treated rats, respectively in (**A**–**E**), where, E: endothelial cells, *Ad*: adventitia. Note, uniform strong immune-reactivity of the α-SMA in SMF of tunica media that are arranged in between the unstained elastic lamellae (arrowhead), areas of weak immune-reactivity (circle), focal areas of sub-endothelial muscular thickening (arrow), wide collapsed lumen (⋆). While (**F**) showed percentage of aortic α-SMA immunohistochemical area. MTX: methotrexate, MTX/AuPs: methotrexate conjugated to AuNPs. Number of rats/group = 10. Data are expressed as means ± SEM. *p* < 0.05 * Significant difference versus normal control, #: Significant difference versus adjuvant-induced arthritis group, € Significant difference versus AuNPs-treated rats, $ Significant difference versus methotrexate-treated rats

Weak immune-reactivity of the tunica media α-SMA was observed with sub-endothelial muscular thickening of the tunica intima in non-treated AIA vessels, which was significantly lower than that of normal control by morphometric analysis. Treatment with MTX-AuNPs restored α-SMA expression in both vessels with focal areas of sub-endothelial muscular thickening that was more evident and significantly higher than all treated and non-treated AIA groups in aorta rather than in femoral artery (Fig. 7A–F for aorta and Fig. S8 A–F for femoral).

## Discussion

Rheumatoid arthritis (RA) is an independent risk factor for CVD (Argnani et al. 2021). The reasons beyond the increased CV morbidity and mortality in RA are likely multifactorial. Growing evidence supports a predominant role for systemic inflammation in promoting endothelial dysfunction and premature atherogenesis, the leading main features for rheumatoid vascular dysfunction (Castañeda et al. 2016). Despite aggressive use of DMARDs, rheumatoid vasculitis remains a resistant rheumatoid manifestation to treat. Reduction of CV risk is a critical goal in the global management of patients with RA besides aggressive treatment of traditional risk factors for atherosclerotic disease (Tanasescu et al. 2009). To overcome the problems commonly encountered with MTX in controlling rheumatoid CV risks, and to get the most out of the gold as effective anti-rheumatic, a nanocarrier drug-delivery system was developed by conjugation of MTX to AuNPs and was compared to free MTX and AuNPs in AIA rat model.

Results of the present study showed evidence of systemic inflammatory response induced by CFA injection; demonstrated by significant increase in serum VCAM-1 and CRP levels in the non-treated AIA rats. This finding was associated with a disturbed lipid profile; noted by significant reduction in HDL-C level and significant increase in LDL-C level, as well as, both atherogenic ratios TC/HDL and LDL/HDL. An impaired vascular responsiveness was expressed by AIA rats’ isolated aortae demonstrated by a reduced relaxation response to cumulative doses of ACh.

The histological and immunohistochemical results expressed disorganization of the vascular architecture of both aorta and femoral arteries with inflammatory cells infiltration of the adventitia and decrease expression of α-SMA in the tunica media. Although premature atherogenesis was more evident in aorta, femoral artery expressed more loss of function that was evident by collapsed lumen filled with sloughed material and red blood cells with thinning out and stretching of the arterial wall. Moreover, H&E revealed the presence of several smaller vessels with disorganized irregular lumens surrounding the femoral artery. This feature could denote an isolated capillaritis, which is a distinguishing feature of rheumatoid associated vascular dysfunction to overcome the relatively reduced function of the main artery (Makol et al. 2015). The perivascular inflammatory infiltrates do not support the diagnosis of overt vasculitis; as it mandates the involvement of the three cell layers of the vessel wall, which usually occurs late in the course of the disease (Bartels and Bridges 2010). However, these findings speculated the occurrence of vascular dysfunction with premature atherosclerotic changes in rats with AIA. The discrepancy in the observed pathology between both vessels is a natural consequence of their different structure and physiological function. Also, these vascular findings were conjoined with histological features of arthritis in the form of thickening of the synovial membrane, congested blood vessels, narrowing of the synovial space, irregular surface of the articular cartilage, as well as bone erosion that were in line with the literature (Eissa et al. 2016; Snekhalatha et al. 2013).

In patients with RA, serum level of VCAM-1 is increased and is reportedly considered a predictive marker for CVD. Generally, VCAM-1 is highly expressed on the injured endothelium, and it is related to the firm adhesion of leukocytes to the endothelium and plays an important role in the development of atherosclerosis by recruiting leukocytes into the sub-endothelial space (Klimiuk et al. 2007). Therefore, the increased serumVCAM-1 in the non-treated AIA rats, not only denotes vascular dysfunction, but also supports the incidence of premature atherosclerosis. In line, Nozaki et al. 2007 reported a significant increase in aortic expression of VCAM-1 in AIA model in rats.

Being a sensitive indicator for systemic inflammation, serum CRP is an independent predictor for preclinical CVD and overall CV mortality in RA patients. In line, CRP independently correlates with preclinical atherosclerotic disease in RA, as assessed by measurements of carotid intima media thickness, carotid plaque, aortic pulse wave velocity, and endothelial cell dysfunction (Fu et al. 2020). Vascular CRP production has modulatory functions by inhibiting endothelial nitric oxide synthase (eNOS) and inducing the expression of adhesion molecules (e.g., VCAM-1) in endothelial cells. Also, CRP has a major role in generation of reactive oxygen species, promoting vasoconstriction and vascular smooth muscle cells migration and proliferation (Erre et al. 2022). Likewise, Petchi et al. 2015 demonstrated a significant increase in serum CRP level in CFA rat model.

The observed lipid profile is in accordance with previous studies on AIA rats, as well as in RA patients (Hendawy et al. 2015; Murunikkara and Rasool 2017; Curtis et al. 2012). Although conflicting results were reported for TC and LDL-C levels across the time course of the RA disease, cardioprotective HDL-C is of particular interest in the context of active inflammation, as its levels dramatically fall and are persistently low, to a far greater extent than the changes seen in other lipid components. Several studies have confirmed an inverse correlation between inflammatory markers, such as CRP and HDL-C (Murunikkara and Rasool 2017). Others reported that RA leads to a more atherogenic lipid profile in the form of increased atherogenic index ‘TC/HDL and LDL/HDL ratios’ that constitutes an important CVD prognostic marker (Parveen et al. 2016; Popa et al. 2012).

The detected impaired vascular reactivity has been previously reported in AIA studies (Nozaki et al. 2007; Haruna et al. 2006). Its impairment is mostly due to the induced rheumatoid chronic inflammatory state that leads to endothelial and smooth muscle dysfunction. The exposure to chemical stress increases endothelial permeability to the inflammatory cells with an increased expression of adhesion molecules and inflammatory cytokines. This would directly decrease eNOS expression and availability explaining the observed herein diminished vascular relaxation response to ACh. Also, penetration of monocytes to the sub-endothelium predisposes to atherogenesis (Su 2015; Mahmoudi et al. 2017).

The histological and the immunohistochemical features of both aorta and femoral arteries in AIA model were not described in the literature, yet the H&E findings of the aorta were in line with a rat model of induced hyperlipidaemia (Moushira et al. 2015). The reduced immune-reactivity of α-SMA, observed herein, and its unusual minor detection in both the sub-endothelium and subintimal layers denote the proliferation of the vascular SMCs and their migration to the intimal layer leading to intimal muscular thickening This significantly indicates vascular remodeling and atherogenesis. Upon arterial wall stress, SMCs change their phenotype by decreasing expression of smooth muscle genes, such as α-SMA and become responsive to chemokines and growth factors that increase their migration and proliferation. Also, endothelial cell derived NO inhibits SMC proliferation and migration, thus, it can be concluded that endothelial cells may, in part, regulate smooth muscle phenotype (Chen et al. 2016). The relationship between endothelial dysfunction, NO depletion, and the expression of α-SMA needs further investigations.

Besides the alteration in SMCs phenotype, the generated inflammatory cytokines create a milieu of increased oxidative stress that can alter vascular architecture through the disorganization of elastin in the lamellae of the medial arterial layer and changes in the composition of the extracellular matrix, thus impairing vascular mechanical function. This could be due to down-regulation of Lysyl oxidase enzyme that is essential to maintain the tensile and elastic features of blood vessels and its deficiency has been implicated in atherosclerosis, fragmentation of elastic fibers, and alterations in endothelial cell functions (Jain et al. 2014). In this context, endothelial dysfunction is considered a preclinical marker of atherosclerosis commonly detected in RA patients (Su 2015; Mahmoudi et al. 2017).

Interestingly, a marked reduction in systemic, and local vascular and joint tissues inflammation, as well as improvement in lipid profiles were observed after two weeks treatment with MTX/AuNPs to the AIA rats. This was evidenced by significant reduction in VCAM-1 and CRP levels compared to the non-treated and treated AIA rats. However, treatment with free MTX rather than non-conjugated AuNPs significantly reduced VCAM-1 and non-significantly reduced CRP denoting a more prominent MTX anti-inflammatory action. Only treatment with MTX/AuNPs significantly raised HDL-C level, though it was non-significantly increased with non-conjugated AuNPs. Both atherogenic ratios were significantly reduced only with MTX/AuNPs treatment. However, LDL-C level was not significantly reduced with both MTX/AuNPs and non-conjugated AuNPs treatments.

These lipid results denote primarily a promising anti-atherogenic potential of AuNPs that became overtly significant by conjugation with MTX. Secondly, free MTX expressed a non-favorable lipid profile that was nearly comparable to the non-treated AIA rats. In RA treatment, a lot of discrepancies involve the effect of MTX on overall atherogenic process, however, a consensus supports the anti-atherogenic potential of MTX and its involvement in the MTX cardioprotective effect in a time-dependent manner (Popkova et al. 2015; Sidhu 2016). This could justify the lack of atherogenic profile improvement, observed herein, by the brief free MTX treatment and highlight the synergistic effect of AuNPs on MTX anti-atherogenic action. This synergistic action involved, also, MTX anti-inflammatory action that became swifter and significantly evident even in the induced ankle joint arthritis. Non-conjugated AuNPs and free MTX treatments showed a non-significant regression of inflammatory process with moderate features of ankle arthritis. As to the unique study that investigated the effectiveness of the MTX/AuNPs in CFA-induced model of arthritis, Chen et al. 2013 reported a better anti-arthritic potential compared to free MTX. In RA treatment, gold compounds have been replaced by more effective and more tolerable anti-inflammatory DMARDs. It seems that AuNPs conjugated to MTX could overwhelm the delayed weak anti-inflammatory action of the previously used gold compounds.

Biologically, isolated aortae from MTX/AuNPs-treated rats expressed a significant enhancement in vascular reactivity even comparable to normal control. The retrieval of vascular function was confirmed histologically by improvement of vascular architecture, reduction in inflammatory cells infiltration, reconstruction of the elastic lamina and the significant regain of α-SMA immune-reactivity, which implies the amelioration of the vascular contractile function. Although both non-conjugated AuNPs and free MTX treatments did significantly enhance aortic vascular reactivity, it was significantly less than MTX/AuNPs treatment. Also, both treatments differently improved vascular architecture. Free MTX treatment, rather than non-conjugated AuNPs, reduced vascular inflammatory infiltration and elastin disorganization, and abolished the feature of isolated capillaritis observed around the femoral artery.

Studies on MTX conjugated to AuNPs focus mainly on the cancer field, where they highlighted the superiority of MTX conjugated to AuNPs over free MTX action. Most studies either declared a better pharmacokinetic profile with a faster and higher accumulation of MTX/AuNPs within the cells or an effective therapeutic outcome with higher cytotoxic effects on several tumor cell lines (Murawala et al. 2014; Van der Heijden et al. 2007). Chen et al. 2007 reported a seven-fold increase in the antineoplastic efficiency of MTX/AuNPs and it has been attributed to what they called the “concentrated effect” of MTX/AuNPs. It is known that one of the factors that determine the effectiveness of any chemotherapeutic agent is not only the diffusion into the target cells, but also, the retention time within the cells in a sufficient concentration to inhibit cell growth and functions. Therefore, it is speculated that AuNPs with their manifest physiochemical properties, such as high tissue permeability, high colloidal stability, and small size, succeeded to rapidly deliver MTX in adequate concentration and retain it within the cell, probably through AuNP-mediated endocytosis (Chen et al. 2007).

Overall, these results indicate that the conjugation of MTX to AuNPs demonstrated a synergistic action over each treatment alone improving MTX therapeutic effectiveness. It combined MTX immunomodulatory action with the potential AuNPs anti-atherogenic action yielding a promising control of the whole features of the arthritis-induced vascular dysfunction. The application of MTX in gold nanocarriers would be a significant advancement in RA treatment to overcome MTX resistance and enhance its therapeutic effectiveness. This would enable reduction of CV events, particularly that mechanisms of MTX resistance in RA involve impaired drug delivery to target cells, defective cellular uptake, and increased drug extrusion (Murawala et al. 2014). Further studies are warranted to unravel AuNPs potentials in increasing MTX cellular retainability, overcoming cellular efflux pumps, and even in enhancing its tolerability.

## Supplementary Information

Below is the link to the electronic supplementary material.

Supplementary file1 (DOCX 3180 KB)

## Data Availability

Datasets generated during and/or analyzed during the current study are available from the corresponding author on reasonable request.

## Abbreviations

RA: Rheumatoid arthritis
CVD: Cardiovascular disease
AuNPs: Gold nanoparticles
DMARDs: Disease modifying anti-rheumatic drugs
CFA: Complete Freund's adjuvant
ED: Endothelial dysfunction
RVD: Rheumatoid vascular dysfunction
