# Supplementary material for: Methotrexate conjugated gold nanoparticles improve rheumatoid vascular dysfunction in rat adjuvant-induced arthritis: gold revival
Source: Inflammopharmacology. 2022 Dec 8;31(1):321–35. doi: 10.1007/s10787-022-01104-w (PMC9958144; doi:10.1007/s10787-022-01104-w)
Supplement: Supplementary file 1 — Supplementary file1 (DOCX 3180 KB) [file 10787_2022_1104_MOESM1_ESM.docx]

**Supplementary Fig. 1 The zeta potential of (*A*) the non-loaded AuNPs and (*B*) the AuNPs conjugated with MTX.** Zeta potential measurements showed a negatively charged surface potential of the non-loaded AuNPs with approximately -34.0 mV that decreased to -25 mV after conjugation with MTX. MTX: methotrexate, AuNPs: gold nanoparticles

**Supplementary Fig. 2 Representative photograph showing clinical signs of inflammation in rats' joints at the onset of arthritis (about 14 days post CFA injection).**

**Supplementary Fig. 3 Serum total cholesterol level in ng/ml in rat’s adjuvant-induced arthritis.** TC: total cholesterol; MTX: methotrexate, MTX/AuNPs: MTX conjugated to gold nanoparticles. Number of rats/group =10. *: Significant difference versus normal control, #: Significant difference versus adjuvant-induced arthritis group, $: Significant difference versus methotrexate-treated rats

**Supplementary Fig. 4 Vascular reactivity in terms of contraction responses to cumulative doses of Norepinephrine (10^-7^ to 10^-4^ mol/L) of endothelium-intact aortic rings** showing a significant difference in contraction response between non-treated AIA aortic rings and normal control and also decreased contraction responses of MTX/AuNPs-treated rings at higher concentration. *: Significant difference versus normal control, #: Significant difference versus adjuvant-induced arthritis group. Data are expressed as Mean ± S.E.M. (n = 6 aortic rings/group). *P*<0.05, by two-way ANOVA for repeated measures. NE: norepinephrine; M: Mole; MTX: methotrexate; MTX/AuNPs: MTX conjugated to gold nanoparticles.

**Supplementary Fig. 5 Representative photomicrographs of H&E-stained sections of rats’ ankle joint.** In (*A&B*) normal control showing thin synovial membrane of the joint capsule **(arrow)**, the joint space **(S)** appears of normal width with preserved smooth hyaline articular surfaces. In (*C&D*) Non-treated AIA showing thickening of the synovial membrane **(double arrows)** with congested blood vessels (BV) and edema. The joint space is narrow with irregular surfaces of the articular hyaline cartilage **(arrowhead)** and areas of bone erosion **(⋆)**

**Supplementary Fig. 6 Representative photomicrographs of rats' femoral tissue sections showing in (*A-E*). H&E stain and in (*F-K*) Orcein stain of normal control, non-treated AIA, AuNPs-, MTX-, and MTX/AuNPs-treated rats, respectively.** E: endothelial cells, m: tunica media, Ad: adventitia, IEL: internal elastic lamella, EEL: external elastic lamella, L: lumen, ACT: adipose connective tissue, V: femoral vein. Note, prominent vasa vasorum **(arrow),** thin stretched internal and external elastic laminae, which appeared interrupted in many parts **(arrowhead)**, smooth muscle fibers showed patchy areas of pale eosinophilic cytoplasm **(circle)**.

**Supplementary Fig. 7 Representative photomicrographs of immunohistochemically stained rats' femoral tissue sections with alpha-smooth muscle actin antibody (HRP/DAB) of normal control, non-treated AIA, AuNPs-, MTX-, and MTX/AuNPs-treated rats, respectively in (*A-F)*,** where, E: endothelial cells, Ad: adventitia. Note, areas of weak immune-reactivity **(circle)**, areas of evident immune-reactivity **(yellow circle)**, smaller vessels surrounding femoral artery with disorganized irregular lumens **(arrowhead)**, smaller vessels with disorganized irregular lumens **(arrow)**. **While (*F*) showed percentage of femoral α-SMA immunohistochemical area.** MTX: methotrexate, MTX/AuPs: methotrexate conjugated to AuNPs. Number of rats/group =10. Data are expressed as means ± SEM. *p*< 0.05 *: Significant difference versus normal control, #: Significant difference versus adjuvant-induced arthritis group.
